# Career decisions and gender: the illusion of choice?

**DOI:** 10.1007/s40037-014-0128-x

**Published:** 2014-06-24

**Authors:** Elspeth J. R. Hill, James A. Giles

**Affiliations:** 1 Division of Plastic and Reconstructive Surgery, Washington University in St Louis, St Louis, MO USA; 2 Department of Neurology, Washington University in St Louis, St Louis, MO USA

In their article published in this issue, Van Tongeren-Alers et al. [1] conducted a systematic review of the evidence for gender differences in speciality preference among medical students. Male students were more likely to be interested in surgery, while female students were more likely to be interested in gynaecology, paediatrics and general practice. Writing in a thought-provoking article in Medical Education, Bleakley made a recent call for a move beyond traditional demography (and indeed biology) to consider gender issues as cultural, in terms of gendered ways of thinking [2]. With such a stance in mind, it is striking that the majority of the studies included in Van Tongeren-Alers et al.’s review framed the problem in terms of ‘career choice’. Given such pronounced gender differences, which Van Tongeren-Alers et al. show hold consistently across countries, can students be truly said to have a ‘choice’ of career? To what extent are their available career options socially and culturally mediated by gender?

We know that certain factors affect students’ career decisions. Chief among these is experience: if students are exposed to a speciality and enjoy it, they are more likely to pursue that career [3]. However, there is evidence that students of different genders experience clinical placements differently. Females have been shown to have differing and more negative experiences of surgery [4], while, equally, males have differing and more negative experiences of gynaecology [5, 6]. The culture of a speciality mediates students’ experiences; in gynaecology, male students are treated differently because of differing expectations within the speciality of male and female students [7]. While experience is all important, some, due to their gender, are better able to access participation in certain specialities as a result of current cultural understandings of gender and the specialities themselves—in other words, because of commonly held gendered discourses of what it is to be male or female, a surgeon or gynaecologist.

Role models are another influence on students’ career decisions, especially during clinical placements [8]. Here, again, gender is an important factor [8–10]. Indeed, female students training in centres with more female surgeons were significantly more likely to show an interest in surgery [11]—potentially explaining how current gender patterns are reproduced over time. Previous work has shown that what students see, hear, and do when encountering a speciality affect whether they can imagine themselves therein [4]. This idea, that of a ‘paradigmatic trajectory’, goes further than role modelling, to encompass how a speciality as a community and culture influences the possible futures students can imagine for themselves. Surgical paradigmatic trajectories are more accessible to male students and deter female students; [4] there is also evidence that paradigmatic trajectories in female-dominated specialities, such as gynaecology, deter male students [5].

The above arguments indicate that social and cultural processes influence career decisions, but can such processes be said to restrict choice? Consideration of discourses may allow deeper exploration of these phenomena. Discourses of masculinity include characteristics of strength, decisiveness, independence, whilst those of femininity include empathy, communication, collaboration [12]. Discursively, surgery is constructed as strongly masculine: its culture is masculine, wherein masculine qualities are valued [13]. Conversely paediatrics is constructed as feminine, highly prizing communication skills and the ability to ‘care’ for children [13].

Poststructuralist feminist critique considers gender not as biological, but as performative [14]—in terms of learned behaviours and beliefs that become embodied; ‘women’ (insofar as the word denotes a common identity) tend to embody discourses considered feminine, and ‘men’ masculine, because they learn to ‘do gender’ [14]. Importantly, this is not to say that men cannot ever embody feminine discourses, nor that individuals can only embody either masculine or feminine discourses. Indeed, men do embody, and operate within, feminine discourses, and women masculine [15]—we have hugely successful male paediatricians and female surgeons as evidence. However, what we do suggest is that in a domain where masculine discourses predominate, such as surgery, feminine qualities have less cultural worth and are therefore more marginalised. It therefore follows that it is harder for those who have constructed a feminine identity for themselves—more usually, though not exclusively, women—to imagine, access and engage in such a practice, especially when they simultaneously compare it (imaginatively or otherwise) to a practice such as paediatrics, which discursively embraces and values those same aspects of their identity.

Different theoretical stances attribute differing degrees of agency—the capacity for self-direction—to individuals ‘making choices’ in the world. The sociocultural stance we adopt above holds that discursive constraints, such as gender, class, ethnicity and sexuality operate to restrict available options; or, to put it another way, the possibilities that are available are largely determined by an individual’s background—or rather their embodied history-in-person—and the conditions of the society in which they operate [16]. Discourses, then, become a ‘highly rigid regulatory frame’ [14].

More individualistic perspectives differ from a sociocultural stance in that individuals become the ‘locus’ of agency; individuals are free to decide their future, taking into account how they see the world. It is towards this end of the epistemological spectrum that we position the literature on ‘career choice’ in medical education, one of whose features as a field is, what Bleakley terms, the ‘widespread uncritical acceptance of andragogy’ [2], which seeks to emphasise the autonomy and ‘self-direction’ of each learner [17]. These individualistic theories are critiqued for their ‘denial of the social world’—not acknowledging the cultural constraints working against those marginalised or less privileged in a society [18, 19].

So, how to proceed? Likely we can all agree on the importance of supporting medical students to navigate the various careers available, and take the best path for themselves. As Van Tongeren-Alers et al. suggest, the method of gender mainstreaming may be useful; [20] it renders issues of gender, and what it means to be male and female, ‘up for negotiation’. We suggest that discussion of what surgery or gynaecology is, in a cultural sense, could also lead to renegotiation and broadening of who should practice them. There may also be space within medical education for further consideration of the influence of gendered discourses in day-to-day practice—of how gender ‘works’ within a culture; this approach allows exploration of demographic inequalities from another angle, theoretically complementing past work.

Studies to identify gender imbalance in medical education are often accompanied by a desire to address the issue. The contemporary social sciences have given us the tools to do so, to move beyond demography and biology in our discussions, in teaching and learning, and in research, and to begin to better address the gender issues that so concern us.
